# Predicting Retention Times of Naturally Occurring Phenolic Compounds in Reversed-Phase Liquid Chromatography: A Quantitative Structure-Retention Relationship (QSRR) Approach

**DOI:** 10.3390/ijms131115387

**Published:** 2012-11-20

**Authors:** Jamshed Akbar, Shahid Iqbal, Fozia Batool, Abdul Karim, Kim Wei Chan

**Affiliations:** 1 Department of Chemistry, University of Sargodha, Sargodha 40100, Pakistan; E-Mails: jamshed.chemist@gmail.com (J.A.); ranashahid313@gmail.com (S.I.); foziaanalytical@yahoo.com (F.B.); drugrelease@yahoo.com (A.K.); 2 Laboratory of Molecular Biomedicine, Institute of Bioscience, Universiti Putra Malaysia, Serdang 43400, Malaysia

**Keywords:** QSRR (quantitative structure-retention relationship), naturally occurring phenolic compounds, artificial neural networks, unsupervised forward selection, reversed phase liquid chromatography

## Abstract

Quantitative structure-retention relationships (QSRRs) have successfully been developed for naturally occurring phenolic compounds in a reversed-phase liquid chromatographic (RPLC) system. A total of 1519 descriptors were calculated from the optimized structures of the molecules using MOPAC2009 and DRAGON softwares. The data set of 39 molecules was divided into training and external validation sets. For feature selection and mapping we used step-wise multiple linear regression (SMLR), unsupervised forward selection followed by step-wise multiple linear regression (UFS-SMLR) and artificial neural networks (ANN). Stable and robust models with significant predictive abilities in terms of validation statistics were obtained with negation of any chance correlation. ANN models were found better than remaining two approaches. HNar, IDM, Mp, GATS2v, DISP and 3D-MoRSE (signals 22, 28 and 32) descriptors based on van der Waals volume, electronegativity, mass and polarizability, at atomic level, were found to have significant effects on the retention times. The possible implications of these descriptors in RPLC have been discussed. All the models are proven to be quite able to predict the retention times of phenolic compounds and have shown remarkable validation, robustness, stability and predictive performance.

## 1. Introduction

Naturally occurring phenolic compounds are widespread among plants; they are synthesized during various metabolic pathways and their concentration varies over a wide range depending upon the plant [1–4]. They have significant importance during the current decade, due to their well-proven antioxidant, anti-aging, antimicrobial and immunomodulatory activities [5,6]. Phenolic compounds provide oxidative stability to foods and beverages, besides contributing health benefits [7–9]. A recent rising interest in the determination of phenolic compounds is mainly due to their potential protective roles against number of diseases associated with oxidative stress or initiated by free radicals, including coronary heart disease, stroke and cancer [10,11]. So the overwhelming beneficial attributes of phenolics requires detailed study of their structure and availability in different food items. For this purpose, separation as well as identification of these compounds is necessary. Numerous analytical approaches have been described in the literature for the analysis of variety of phenolics [12–15]. In this context, reversed-phase liquid chromatography-mass spectrometry is considered a practically state-of-the-art technique; as reversed-phase liquid chromatography (RPLC) provides better separation and mass spectrometry (MS) gives sensitive detection and confirms structures of compounds [16].

Quantitative structure-retention relationships (QSRRs) have gained wide attention in the area of separation science recently. These models are based on the relationship between structures and properties of compounds. Retention times of different compounds can be predicted from their formulae and even unknown compounds can be identified by using this method. In general, QSRR models attempt to predict the retention time of a molecule by characterizing it with a series of molecular descriptors. These models can effectively be used for the prediction of molecular structures, determination of retention times of new analytes and to understand the separation mechanism for a chromatographic system [17]. Several QSRRs have been developed to predict the retention times of different analytes on different systems [18–24]. Applications and implications of QSRR methodology in chromatography has recently been thoroughly reviewed and emphasized [25,26]. No comprehensive report describing the QSRR study of phenolic compounds from natural sources has been presented so far. Naturally occurring phenolic compounds belong to varied classes, have a range of simple to complex structures and therefore, need a compact statistical approach of QSRRs. The aim of this study is to develop statistically significant QSRR models, based on structural descriptors, for the prediction of retention times of naturally occurring phenolic compounds in RPLC. The approach consists of reduction of large descriptor pool to the most relevant descriptors with minimum multicollinearity and redundancy. The SMLR and UFS-SMLR have been used as supervised and unsupervised-supervised algorithms to reduce the descriptor pool. The selected descriptors are then used to generate ANN models with enhanced statistical significance. The study has generated reasonably stable, robust, and predictive models, which could provide an effective tool for predicting and analyzing the retention behavior of naturally occurring phenolic compounds in RPLC.

## 2. Results and Discussion

A total of 1519 descriptors were calculated from optimized structures of phenolics by use of MOPAC2009 and DRAGON version 3 softwares (Table 1). The descriptors were initially filtered by removing those with zero values, constant values for 50% of the compounds and variance less than 0.0005. This pretreatment left a total of 915 descriptors in the data, which were subsequently used for model generation.

For QSRR development, data set of 39 phenolic compounds [27] was randomly split into a training set of 30 molecules and an external validation set of nine molecules. For the purpose of model generation, retention times (RT) were used as response variables.

### 2.1. Stepwise Multiple Linear Regression Model (SMLR Model)

The 915 descriptors, survived after initial filtration, were used to construct models by SMLR method using a sufficiently stringent criterion (*F* = 6 to enter, *F* = 3 to remove) in order to keep less number of descriptors in the model so as to avoid multi-collinearity. The five descriptor model based on training set for predicting retention times of phenolics is

(1) $$\begin{array}{c} \text{RT}=5.527(\pm 0.584)\;\text{HNar}-3.462(\pm 0.348)\;\text{GATS}2\text{v}+4.161(\pm 0.320)\;\text{DISPe}-\\ 1.386(\pm 0.305)\;\text{Mor}32\text{e}+1.634(\pm 0.514)\;\text{Ke}-4.451(\pm 1.012)\\ \left(N=30,R^{2}=0.962,{\text{PRESS}}_{\text{int}}=0.062,{Q^{2}}_{\text{int}}=0.941,{\text{PRESS}}_{\text{ext}}=1.929,{Q^{2}}_{\text{ext}}=0.760\right) \end{array}$$

Equation 1 showed good stability as indicated by internal and external validation coefficients of determination. All the five descriptors exhibited very weak or negligible correlations with one another (Table 2). Of all the descriptors, Ke, which appeared in step five of SMLR, showed somewhat more correlations with others, though not much significant, therefore, dropping this from the equation resulted in another equation with less number of descriptors and still of good statistical quality (Equation 2).

Dropping step four descriptor Mor32e also resulted in a good model but it was comparatively poor in terms of external validation (Equation 3). The four descriptor model (Equation 2) was selected as an optimal model. The relative significance of descriptors in this model was ascertained by test statistics in Minitab 15. The corresponding *T*- and *p*-values for the individual terms in Equation 2 are: HNar, *T* = 11.19, *p* < 0.001; GATS2v, *T* = −8.31, *p* < 0.001; DISPe, *T* = 11.07, *p* < 0.001; Mor32e, *T* = −3.71, *p* = 0.001. Low *p*-values indicate that these terms are significant in predicting retention times.

(2) $$\begin{array}{c} \text{RT}=0.771(\pm 0.581)\;\text{HNar}-0.326(\pm 0.348)\;\text{GATS}2\text{v}+0.467(\pm 0.374)\;\text{DISPe}-0.115(\pm 0.356)\;\text{Mor}32\text{e}-0.686(\pm 1.092)\\ \left(N=30,R^{2}=0.946,{\text{PRESS}}_{\text{int}}=0.080,{Q^{2}}_{\text{int}}=0.924,{\text{PRESS}}_{\text{ext}}=1.847,{Q^{2}}_{\text{ext}}=0.770\right) \end{array}$$

(3) $$\begin{array}{c} \text{RT}=7.574(\pm 0.615)\;\text{HNar}-2.242(\pm 0.367)\;\text{GATS}2\text{v}+3.792(\pm 0.442)\;\text{DISPe}-0.740(\pm 1.149)\\ \left(N=30,R^{2}=0.917,{\text{PRESS}}_{\text{int}}=0.116,{Q^{2}}_{\text{int}}=0.890,{\text{PRESS}}_{\text{ext}}=2.683,{Q^{2}}_{\text{ext}}=0.666\right) \end{array}$$

**Figure 1:**
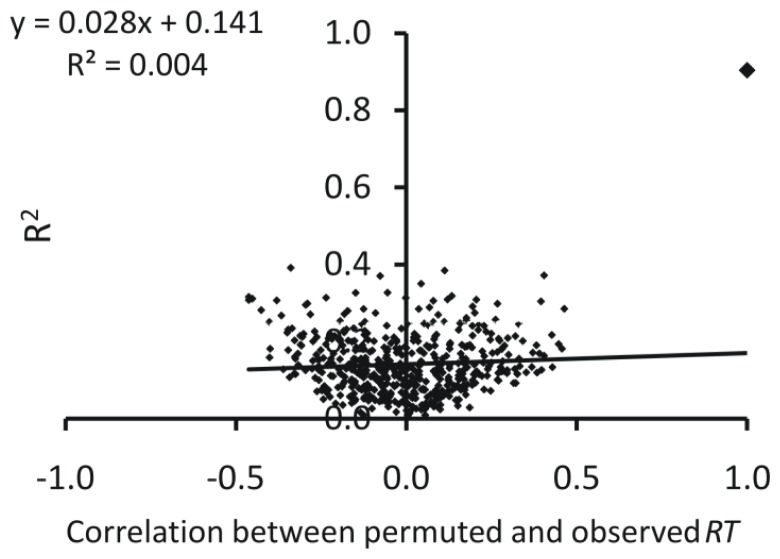
Representative *y*-scrambling plot (SMLR model).

*y*-Scrambling result was also encouraging for Equation 2 (Figure 1), where most of the scrambled models have statistical parameters clustered around zero in a symmetrical way, indicating that the scrambled models are of very low quality. Intercept value of the plot between *R*^2^ values of the scrambled models and correlation of observed and permuted responses was very low (0.141). This establishes the stability of model and eliminates possibility of any chance correlation.

Unsupervised Forward Selection-Stepwise Multiple Linear Regression Model (UFS-SMLR Model)

The 915 descriptors left after pretreatment were subjected to UFS algorithm with *R*^2^_max_ = 0.90, that decreased the data set to only 22 linearly independent descriptors with minimum multi-collinearity and redundancy (Table 3).

The SMLR method applied to UFS-selected descriptors produced a six descriptors model (Equation 4). This model is quite good in terms of the entire applied statistical criterion, though, less significant than SMLR model as indicated by the PRESS and co-efficient of determination statistics.

(4) $$\begin{array}{c} \text{RT}=29.480(\pm 2.997)\;\text{Mp}+0.208(\pm 0.056)\;\text{IDM}+0.208(\pm 0.033)\;\text{DISPm}-2.704(\pm 1.121)\;\text{Mor}22\text{v}-\\ 1.650(\pm 0.445)\;\text{Mor}28\text{e}+4.819(\pm 1.610)\;\text{HATS}7\text{p}-18.788(\pm 1.974)\\ \left(N=30,R^{2}=0.912,{\text{PRESS}}_{\text{int}}=0.155,{Q^{2}}_{\text{int}}=0.852,{\text{PRESS}}_{\text{ext}}=2.293,{Q^{2}}_{\text{ext}}=0.715\right) \end{array}$$

**Figure 2:**
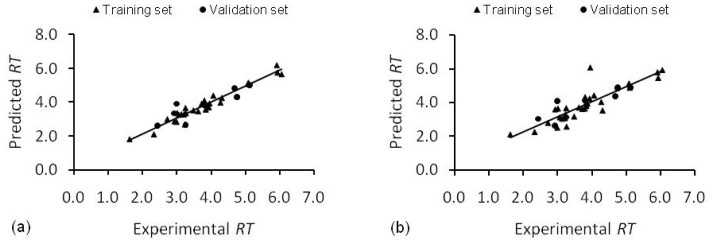
Experimental and predicted retention times (RT) for training and validation sets. (**a**) SMLR model (**b**) UFS-SMLR model.

Selecting a five descriptor model (Equation 5), after removal of step six descriptor, Mor28e, also showed a good predictive ability. The *T*- and *p*-values for individual terms in Equation 5 are: Mp, *T* = 9.30, *p* < 0.001; IDM, *T* = 9.30, *p* < 0.001; DISPm, *T* = 5.08, *p* = 0.003; Mor22v, *T* = −2.64, *p* = 0.014; Mor28e, *T* = −3.33, *p* = 0.003. The Mor22v descriptor has slightly higher *p*-value, however, all terms appeared to be significant in predicting retention times. The predictions made by Equations 2 and 5 are given in Figure 2.

(5) $$\begin{array}{c} \text{RT}=31.412(\pm 3.376)\;\text{Mp}+0.216(\pm 0.065)\;\text{IDM}+0.186(\pm 0.037)\;\text{DISPm}-3.350(\pm 1.270)\;\text{Mor}22\text{v}-\\ 1.707(\pm 0.513)\;\text{Mor}28\text{e}+19.576(\pm 2.258)\\ \left(N=30,R^{2}=0.877,{\text{PRESS}}_{\text{int}}=0.189,{Q^{2}}_{\text{int}}=0.820,{\text{PRESS}}_{\text{ext}}=1.906,{Q^{2}}_{\text{ext}}=0.763\right) \end{array}$$

*y*-Scrambling result for UFS-SMLR was found similar to SMLR model, though slightly of less quality with *R*^2^ value 0.172.

### 2.2. Artificial Neural Network

The network architecture and validation statistics are given in Table 4.

**Figure 3:**
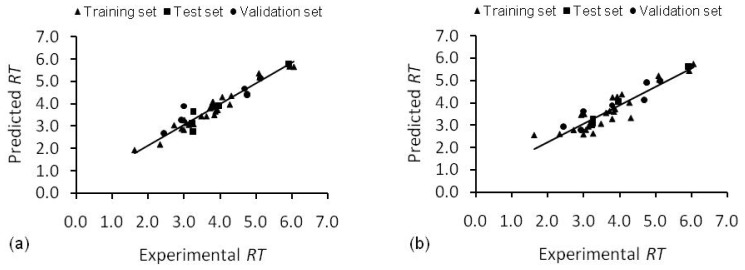
Experimental and predicted retention times (RT) for training, test and validation sets. (**a**) SMLR-ANN model (**b**) UFS-SMLR-ANN model.

In this study, the whole data has been divided into three sets: training, test and validation sets. A test set is used for early stopping of training in order to avoid overfitting. Sometimes the test data alone may not provide an evidence of a good generalization an ANN e.g., it can be just a coincidence. To make sure that this is not the case, another validation set was used. This puts an extra check on the performance and generality of ANN. To make things clearer, the training, test and validation sets have been marked in Table 5. ANN models are better than both SMLR and UFS-SMLR models. Though, SMLR model is comparable to SMLR-ANN model, nevertheless, the real strength of artificial neural network mapping technique was observed for UFS-SMLR-ANN model, which showed considerably better prediction ability than the simple UFS-SMLR model as depicted by *Q*^2^_ext_. In ANN models, the global sensitivity analysis was performed which ranked the descriptors of SMLR-ANN model as HNar > DISPe > GATS2v > Mor32e and UFS-SMLR-ANN model as Mp > DISPm > Mor22v > IDM > Mor28e. The predictions of SMLR-ANN and UFS-SMLR-ANN are presented in Figure 3.

### 2.3. Interpretation of the Models

In case of selected SMLR model (Equation 2), HNar and GATS2v are 2D descriptors derived from molecular graph. HNar is the Narumi harmonic topological index related to molecular branching and represents the number of non-hydrogen atoms divided by the reciprocal vertex degree [28]. Its positive coefficient suggests that increase in HNar leads to an increase in RT. GATS2v is the Geary autocorrelation-lag2 weighted by atomic van der Waals volumes. The autocorrelation descriptors show the distribution of a certain property in the topological structure [29]. The GATS2v descriptor shows the distribution of atomic volume at a distance of two bonds in the topological structure of molecule. The negative coefficient of GATS2v is an indicative of decrease in RT with an increase in lag2 autocorrelations of atomic volumes on molecular graph. The descriptors DISPe and Mor33e are derived from three dimensional structures of the molecules. DISPe is the d COMMA2 value weighted by atomic senders on electronegativities and it represents the displacement between the geometric and the electronegativity centers of the molecule [30]. The positive coefficient of DISPe indicates that molecules with increased displacement between the geometric and the electronegativity centers will take more time to elute. Mor32e is the 3D-MoRSE signal-32, weighted by atomic Sanderson electronegativities. The 3D-MoRSE signals give three dimensional molecular representation of structure based on electron diffraction and contain information on mass distribution and branching within a molecule [31]. The negative coefficient for Mor32e suggests an inverse relation with RT. It follows, therefore, that molecule with more branching, less lag-2 autocorrelation of atomic volumes, enhanced displacement between the geometric and the electronegativity centers and low value of Mo32e descriptor will have more retention times in RPLC.

For UFS-SMLR selected model (Equation 5), Mp is a constitutional descriptor while IDM is a 2D topological descriptor. Mp is the mean atomic polarizability scaled on carbon atom, IDM is the mean information content on the distance magnitude. DISPm, and 3D-MoRSE signals are 3D descriptors. DISPm is the d COMMA2 value weighted by atomic masses, Mor22v and Mor28e are the 3D-MoRSE signals, 22 and 28, weighted by atomic van der Waals volumes and atomic Sanderson electronegativities, respectively. UFS-SMLR model also emphasized the importance of topological descriptor (IDM), atomic volume and atomic electronegativity based 3D descriptors of molecules for the retention behavior of phenolic compounds, as was observed in SMLR selected descriptors. Despite the weighing schemes, the behavior of three dimensional descriptors was similar in both approaches. 3D-MoRSE descriptors related negatively and 3D geometrical DISP descriptors related positively with the retention times in both types of models. This corresponds to similar effects of 3D descriptors in developed QSRRs. A positive coefficient for Mp is an indication of increase in retention time with increase in mean atomic polarizability. In phenolics, oxygen atom is largely present either as hydroxyl group (independent or as a part of carboxyl group) or as ether linkage. Based on the relative nature of carbon, hydrogen and oxygen, it is expected that a decrease in number of hydroxyl groups increases the Mp value. It therefore, suggests that molecules with more hydroxyl group will have low values of Mp and hence they are eluted earlier with the polar mobile phase due to greater number of polar hydroxyl groups in them and hence have less retention times. This behavior can be well observed in case of Gallic acid, Gentisic acid and Salicylic acid (Table 5, Table S1 supplementary data) containing four, three and two hydroxyl groups with Mp values 0.64, 0.65 and 0.67, respectively. The other descriptor IDM also relates directly to RT suggesting an increase in RT with increase in its value. This descriptor provides mean information content on distance magnitude and it is expected to increase with increase in number of atoms in a molecule. Another descriptor DISPm is an indicative of conformational features of molecules. It is generally suggested that rigid molecules have low values of DISPm [29]. This descriptor relates directly to RT which suggests that rigid molecule will have less retention time. The foregoing discussion revealed that generally molecules with more hydroxyl groups, less number of atoms, rigidity and high values of 3D-MoRSE descriptors are eluted faster than others. Mathematical detail of the molecular descriptors is available in the Handbook of Molecular Descriptors [32].

**Figure 4:**
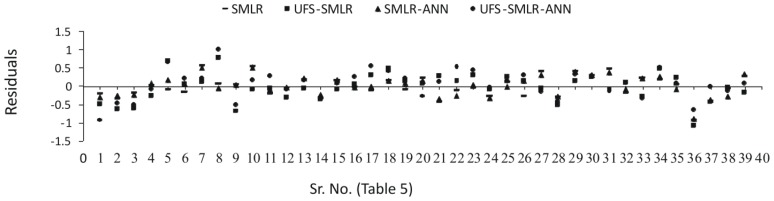
Residual plot for QSRR models.

Quantum mechanical descriptors failed to make any impact, whatsoever, on the models. Equations 2 and 5 and optimal artificial neural networks (Table 4) were used to predict the retention times of naturally occurring phenolic compounds. The predicted results are presented in Table 5, Figures 2 and 3 and residual plot for the developed models is presented in Figure 4.

## 3. Experimental Section

### 3.1. Data for Retention Times of Phenolic Compounds

Data used to generate structure-retention relationship of phenolic compounds were obtained from a recently developed sharp method of their analysis in RPLC-MS system [27]. Briefly, the compounds were separated by gradient elution, using a reversed-phase C_18_ analytical column (50 × 2 mm, 2.5 μm particle size; Phenomenex Synergi Fusion-RP100A) with a C_18_ guard column (4 × 2 mm; Phenomenex Fusion-RP) maintained at 35 °C. The mobile phase used was deionized water (A) and acetonitrile (B); each containing 0.1% (v/v) formic acid in a linear gradient from 1% to 100% B during 9.5 min.

### 3.2. Descriptor Computation

Three dimensional structures of phenolic compounds, created by using Chemsketch, were optimized by the use of semi-empirical PM6 Hamiltonian with eigen vector following (EF) algorithm implemented in MOPAC2009 software [33]. Calculation of numerical descriptors from optimized geometries was performed usingMOPAC2009 and DRAGON, version 3 [34] softwares. Total number of calculated descriptors was 1519. Molecular weight (MW) descriptor was duplicated in both the softwares, therefore, MW only from MOPAC2009 was used in this study. Dragon was used to compute 1497 descriptors divided into 18 logical blocks and 23 descriptors were obtained from MOPAC2009 (Table 1).

### 3.3. Feature Selection and Model Generation

Step-wise multiple linear regression (SMLR) and unsupervised forward selection followed by step-wise multiple linear regression (UFS-SMLR) was used for feature selection. UFS is a technique to remove redundant and multi-collinear descriptors from the data set [35]. UFS was performed with ufs-1.8, obtained from the Centre for Molecular Design (CMD), University of Portsmouth, using *R*^2^_max_ = 0.9. The subset of descriptors produced by UFS was later used to develop model by SMLR method. Before applying the regression method, all the data were standardized to zero mean and unit variance in order to avoid any biased nature of the calculated descriptors, which may lead to series errors in generation and application of the models. The standardized data were subjected to SMLR method for model generation.

ANN is a powerful multivariate data analysis technique, capable of both linear and non-linear modeling and has been widely used in modeling structure-property relationships [22,36,37]. An ANN mathematical model mimics the human brain intelligence system and consists of various interconnecting neurons organized in a sequential manner into an input layer, one or more hidden layers and an output layer. Each interconnection of the neurons has some numerical value (weight) associated with it. The signals are transmitted from the input layer to output layer through the neurons. The whole network is first trained on some data by adjusting the interconnection weights and is subsequently used to make predictions for external data. In the present study, optimal number of descriptors, selected by SMLR and UFS-SMLR techniques, was entered as continuous input signals into ANNs and output was the response variable RT. 500 ANNs were trained in both cases by the use of Statistica 8.0 automated artificial neural network implementation. Multilayer perceptrons (MLP) type network with feed-forward topology, Broyden-Fletcher-Goldfarb-Shanno (BFGS) algorithm and normal randomization were used for ANNs training and sum-of-squares error function was used to test their performances. Identity, logistic, exponential and tanh activation functions both for hidden and output layer and number of hidden units from 3 to 8 were used in ANNs building. The models, exhibiting least external validation errors, were selected as optimal models. In ANNs building process, an early stopping technique was employed to avoid over-training of the ANN models. For this purpose, the training set was further sub-divided randomly into a subset of 25 molecules for training the ANNs and a subset of five molecules as a test set to avoid over-fitting. In the development of both SMLR descriptors based ANN (SMLR-ANN) and UFS-SMLR descriptors based ANN (UFS-SMLRANN), same subsets of training set were used. Further, for external validation of all the models, same external validation set of nine molecules was used.

### 3.4. Model Validation

Model validation is a requisite to assess the applicability of generated models. Several techniques are in use in chemometrics [38–41]. In the present study, models were validated both internally as well as externally and any chance correlation was tested by the use of a *y*-scrambling technique: a method frequently used for this purpose. Internal validation was performed by leave-one-out cross validation and external validation by applying the model on external validation set of nine molecules. The statistical quality of the model was judged by considering the sum of squares of prediction errors and the validation correlation coefficients *Q*^2^_int_ & *Q*^2^_ext_ for internal and external validation respectively (Equations 6 and 7, respectively).

(6) $$\text{PRESS}=\sum_{i=1}^{n}{\left({\hat{y}}_{i}-y_{i}\right)}^{2}$$

(7) $$Q^{2}=1-\frac{\sum_{\text{i}=1}^{\text{n}}{\left({\hat{y}}_{i}-y_{i}\right)}^{2}}{\sum_{i=1}^{n}{\left(y_{i}-{\bar{y}}_{\text{train}}\right)}^{2}}$$

where *ŷ_i_* is the predicted value, *y_i_* is the observed value for *i*th case in training or validation set as the case may be, and *y̆*_train_ is the mean of the training set. In above expressions, mean of the training set was used in order to have same standard reference for both internal and external validation statistics. However, using mean of validation set made almost no difference in the present study. For example, in case of SMLR model, *Q*^2^_ext_ using training set mean was 0.769, while using validation set mean, it was 0.770. *y*-Scrambling was performed 500 times for the models in order to establish the stability of model and to negate any chance correlation. The statistical quality parameters of the scrambled models were compared with those of the original models. Performance of the selected ANN models was judged by the *Q*^2^_ext_ statistics. All the statistical calculations were performed using Statistica 8.0 and MS Excel^®^ 2007.

## 4. Conclusions

SMLR, UFS-SMLR and ANN directed QSRR models have successfully been developed for predicting the retention times of naturally occurring phenolic compounds in the RPLC system. ANN models are more authentic in prediction of retention times of phenolics in RPLC than the other two approaches. SMLR model is comparable to SMLR-ANN, however, UFS-SMLR model was found less predictive than others. The models identified Mp, IDM, HNar, DISP, GATS2v and 3D-MoRSE (signals 22, 28 and 32), descriptors responsible for the retention of phenolic compounds. These descriptors signify the importance of branching, size, hydroxyl groups and 3D geometric, electronegativity and mass distribution features within phenolics. The models were found predictive and robust.

## Supplementary Materials



## Figures and Tables

**Table 1:** Descriptors used in the study.

Method/Type	Descriptors
**MOPAC2009/Quantum mechanical**	Total energy, electronic energy, core-core repulsion, dielectric energy, dipole moment, ionization energy, energies of highest occupied molecular orbital (*E*_HOMO_) and lowest unoccupied molecular orbitals (*E*_LUMO_), difference of *E*_LUMO_ and *E*_HOMO_, hardness, softness, molecular mass, cosmo area, cosmo volume. Logarithmic transformations of dipole moment, ionization energy, *E*_LUMO_, difference of *E*_LUMO_ and *E*_HOMO_, hardness, softness, molecular mass, cosmo area and cosmo volume.
**DRAGON/18 blocks of descriptors**	Constitutional, topological, molecular walk counts, BCUT, Galvez topological charge indices, 2D autocorrelations, charge descriptors, aromaticity indices, Randic molecular profiles, geometrical, RDF, 3D-MoRSE, WHIM, GETAWAY, functional groups, atom-centered fragments, empirical and properties.

**Table 2:** Correlations of the descriptors in SMLR model.

	HNar	GATS2v	DISPe	Mor32e	Ke
**HNar**	1.0000				
**GATS2v**	−0.0482	1.0000			
**DISPe**	0.1253	0.1566	1.0000		
**Mor32e**	−0.4053	−0.4069	0.0784	1.0000	
**Ke**	0.4727	0.4644	0.1360	−0.3608	1.0000

**Table 3:** UFS selected descriptors with *R*^2^_max_ = 0.90.

Descriptors	Name	Type
IDM	Mean information content on the distance magnitude	Topological
MATS6p	Moran autocorrelation-lag6/weighted by atomic poloarizabilities	2D-autocorrelations
Mp	Mean atomic polarizability (scaled on carbon atom)	Constitutional
E1e	1st component accessibility directional WHIM index/weighted by atomic Sanderson electronegativities	WHIM
MATS6e	Moran autocorrelation-lag6/weighted by atomic Sanderson electronegativities	2D-autocorrelations
Mor30m	3D-MoRSE-signal 30/weighted by atomic masses	3D-MoRSE
AROM	Aromaticity	Aromatic indices
E3u	3rd component accessibility directional WHIM index/unweighted	WHIM
Mor22v	3D-MoRSE-signal 22/weighted by atomic volume	3D-MoRSE
Mor28e	3D-MoRSE-signal 28/weighted by atomic Sanderson electronegativities	3D-MoRSE
Mor29m	3D-MoRSE-signal 29/weighted by atomic masses	3D-MoRSE
DISPm	d COMMA2 value/weighted by atomic masses	Geometrical
PJI3	3D petijean shape index	Geometrical
G3s	3rd component accessibility directional WHIM index/weighted by atomic electrotopological states	WHIM
MATS5e	Moran autocorrelation-lag5/weighted by atomic Sanderson electronegativities	2D-autocorrelations
PJI2	2D petijean shape index	Topological
SIC4	Structural information content (neighbourhood symmetry of 4-order)	Topological
E2p	3rd component accessibility directional WHIM index/weighted by atomic poloarizabilities	WHIM
Mor12e	3D-MoRSE-signal 12/weighted by atomic Sanderson electronegativities	3D-MoRSE
IVDE	Mean information content vertex degree equality	Topological
SPI	Superpendentic index	Topological
HATS7p	Leaverage-weighted autocorrelation of lag 7/weighted by atomic poloarizabilities	GETAWAY

**Table 4:** Architecture and validation statistics of the optimal ANNs.

	SMLR-ANN	UFS-SMLR-ANN
No. of neurons in the input layer	4	5
No. of neurons in the hidden layer	6	5
No. of neurons in the output layer	1	1
Hidden weight decay	0.01	0.01
Output weight decay	0.01	0.01
Hidden activation function	Tanh	Exponential
Output activation function	Tanh	Logistic
PRESS_ext_	1.4841	1.1021
*Q*^2^_ext_	0.8145	0.8622
Training error	0.0013	0.0047
Test error	0.0021	0.0009
Validation error	0.0042	0.0031

**Table 5:** Experimental and predicted retention times (RT) of naturally occurring phenolic compounds.

Sr No.	Compound	Experimental RT (min)	Predicted RT (min)			

			SMLR	UFS-SMLR	SMLR-ANN	UFS-SMLR-ANN
1	Gallic acid	1.63	1.82	2.12	1.94	2.54
2	Gentisic acid	3.02	3.36	3.65	3.28	3.49
3	Protocatechuicacid b	2.43	2.61	3.04	2.67	2.94
4	Salicylic acid a	3.96	3.93	4.23	3.89	4.04
5	Syringic acid	3.27	3.36	2.58	3.10	2.61
6	Vanillic acid	3.14	3.29	3.05	3.07	2.93
7	2,4-Dihydroxybenzoic acid b	3.26	2.67	3.13	2.76	3.05
8	3-Methoxybenzoic acid	4.32	4.25	3.53	4.37	3.31
9	4-Hydroxybenzoic acid	2.94	2.88	3.60	2.90	3.45
10	Caffeicacid a	3.24	2.69	3.31	2.74	3.08
11	Chlorogenic acid	3.07	3.26	3.13	3.16	2.78
12	Ferulicacid b	3.80	3.84	4.11	3.84	3.89
13	*m*-Coumaric acid	3.88	3.69	3.94	3.67	3.71
14	*o*-Coumaric acid	4.07	4.39	4.42	4.31	4.37
15	*p*-Coumaric acid	3.63	3.47	3.70	3.45	3.54
16	Sinapic acid	3.85	3.86	3.80	3.89	3.59
17	*trans-*Cinnamicacid b	4.69	4.80	4.38	4.69	4.14
18	Dihydrocaffeic acid	3.00	2.84	2.52	2.85	2.57
19	Homovanillicacid a	3.22	3.29	3.08	3.14	3.00
20	DOPAC	2.34	2.11	2.27	2.19	2.59
21	4-hydroxyphenylacetic acid b	2.92	3.34	2.64	3.28	2.79
22	Ellagic acid	3.80	3.90	3.65	4.07	3.27
23	Vanillin	3.49	3.52	3.18	3.45	3.05
24	Tyrosol	2.73	3.00	2.80	3.05	2.77
25	Apigenin b	5.14	5.01	4.88	5.16	4.99
26	Chrysin a	5.92	6.18	5.78	5.77	5.62
27	Luteolin b	4.76	4.33	4.82	4.45	4.90
28	Luteolin-7-*O*-glucoside	3.81	4.10	4.32	4.10	4.24
29	Kaempferide	6.06	5.65	5.91	5.66	5.74
30	Myricetin	4.28	3.98	4.03	3.98	4.00
31	Quercetin b	4.76	4.28	4.87	4.39	4.89
32	Rutin	3.73	3.91	3.62	3.82	3.62
33	Hesperidin	3.94	3.71	4.23	3.73	4.26
34	Isosakuranetin	5.94	5.74	5.45	5.68	5.43
35	Naringenin	5.11	5.05	4.87	5.20	5.04
36	(+)-Catechin b	2.99	3.91	4.07	3.89	3.63
37	(−)-Epicatechin a	3.26	3.66	3.67	3.63	3.28
38	Genistein	5.09	5.15	5.12	5.37	5.21
39	(+)-Taxifolin	3.85	3.57	4.02	3.51	3.78

For ANN models, compounds labelled with letter

a represent molecules in the test set, while those with

b represent molecules in the validation set and unlabelled compounds are in training set.
