# Supplementary material for: Predicting Retention Times of Naturally Occurring Phenolic Compounds in Reversed-Phase Liquid Chromatography: A Quantitative Structure-Retention Relationship (QSRR) Approach
Source: Int J Mol Sci. 2012 Nov 20;13(11):15387–400. doi: 10.3390/ijms131115387 (PMC3509648; doi:10.3390/ijms131115387)
Supplement: Supplementary file 1 [file ijms-13-15387-s001.pdf]

Supplementary Materials

Table S1. Values of descriptors selected in SMLR and UFS-SMLR models.

Sr. No.	Compound	HNar	GATS2v	DISPe	Mor32e	Mp	IDM	DISPm	Mor22v	Mor28e
1	Gallic acid	1.565	1.177	0.065	−0.351	0.640	5.878	2.477	–0.025	0.126
2	Gentisic acid	1.610	1.168	0.374	–0.304	0.650	5.624	6.421	–0.073	–0.093
3	Protocatechuicacid	1.610	1.168	0.210	–0.253	0.650	5.606	4.129	–0.032	–0.068
4	Salicylic acid	1.667	1.157	0.464	–0.151	0.670	5.339	6.808	–0.035	–0.130
5	Syringic acid	1.615	0.668	0.066	–0.155	0.630	6.341	1.995	–0.072	–0.228
6	Vanillic acid	1.636	0.859	0.144	–0.168	0.640	5.867	4.871	–0.046	–0.117
7	2,4-Dihydroxybenzoic acid	1.610	1.168	0.238	–0.212	0.650	5.613	3.879	–0.082	–0.048
8	3-Methoxybenzoic acid	1.692	0.850	0.306	–0.090	0.650	5.603	5.865	–0.038	–0.151
9	4-Hydroxybenzoic acid	1.667	1.157	0.186	–0.231	0.670	5.306	3.998	–0.058	–0.031
10	Caffeic acid	1.660	1.205	0.160	–0.301	0.660	6.060	2.939	–0.009	–0.161
11	Chlorogenic acid	1.676	1.278	0.264	–0.488	0.630	7.975	2.307	–0.015	–0.421
12	Ferulicacid	1.680	0.946	0.287	–0.114	0.650	6.286	6.828	–0.047	–0.283
13	*m*-Coumaric acid	1.714	1.196	0.336	–0.226	0.670	5.825	5.429	–0.020	–0.078
14	*o*-Coumaric acid	1.714	1.196	0.499	–0.245	0.670	5.835	6.865	–0.091	–0.062
15	*p*-Coumaric acid	1.714	1.196	0.272	–0.256	0.670	5.810	3.809	–0.066	–0.030
16	Sinapic acid	1.655	0.773	0.210	–0.112	0.640	6.701	5.944	–0.080	–0.267
17	*trans*-Cinnamic acid	1.784	1.186	0.504	–0.170	0.680	5.560	6.354	0.011	–0.147
18	Dihydrocaffeic acid	1.660	1.259	0.205	–0.397	0.640	6.060	2.890	0.042	–0.169
19	Homovanillic acid	1.660	0.960	0.168	–0.196	0.640	6.085	5.035	–0.082	–0.016
20	DOPAC	1.636	1.222	0.040	–0.394	0.640	5.842	0.416	–0.086	–0.071
21	4-hydroxyphenylacetic acid	1.692	1.207	0.255	–0.343	0.650	5.569	2.793	–0.077	0.105
22	Ellagic acid	1.833	0.869	0.001	–0.135	0.710	7.684	0.027	0.138	0.164
23	Vanillin	1.692	0.817	0.099	–0.115	0.650	5.607	4.086	–0.055	–0.109
24	Tyrosol	1.765	1.169	0.077	–0.201	0.640	5.283	4.618	–0.008	–0.145

Table S1. *Cont.*

Sr. No.	Compound	HNar	GATS2v	DISPe	Mor32e	Mp	IDM	DISPm	Mor22v	Mor28e
25	Apigenin	1.875	1.061	0.220	–0.501	0.700	7.347	3.675	–0.141	0.158
26	Chrysin	1.932	1.057	0.458	–0.347	0.710	7.208	6.346	–0.093	–0.003
27	Luteolin	1.826	1.064	0.141	–0.481	0.690	7.493	2.822	–0.150	–0.047
28	Luteolin-7-*O*-glucoside	1.811	1.131	0.115	–0.605	0.650	8.709	5.401	–0.072	–0.209
29	Kaempferide	1.833	0.901	0.377	–0.350	0.680	7.626	8.482	–0.138	–0.259
30	Myricetin	1.747	1.070	0.149	–0.596	0.680	7.774	2.566	–0.108	0.157
31	Quercetin	1.784	1.067	0.133	–0.680	0.690	7.641	3.152	–0.111	–0.095
32	Rutin	1.779	1.165	0.135	–0.631	0.630	9.590	6.790	–0.115	0.182
33	Hesperidin	1.804	1.116	0.168	–0.148	0.620	9.554	5.027	–0.174	–0.440
34	Isosakuranetin	1.881	0.949	0.345	–0.387	0.670	7.478	8.139	–0.100	–0.306
35	Naringenin	1.875	1.094	0.204	–0.652	0.680	7.347	3.722	–0.207	–0.069
36	(+)-Catechin	1.826	1.137	0.115	–0.405	0.660	7.495	1.878	0.001	–0.557
37	(–)-Epicatechin	1.826	1.137	0.082	–0.315	0.660	7.495	1.225	–0.014	–0.364
38	Genistein	1.875	0.996	0.169	–0.623	0.700	7.347	2.274	–0.178	–0.062
39	(+)-Taxifolin	1.784	1.111	0.096	–0.359	0.670	7.641	2.375	–0.047	–0.177

© 2012 by the authors; licensee MDPI, Basel, Switzerland. This article is an open access article distributed under the terms and conditions of the Creative Commons Attribution license (http://creativecommons.org/licenses/by/3.0/).
